# Editorial: Delay in cancer diagnosis and factors affecting outcomes

**DOI:** 10.3389/fpubh.2024.1442764

**Published:** 2024-07-12

**Authors:** Sara Haddadi, Mehdi Dehghani, Gina D'Amato

**Affiliations:** ^1^Division of Hematology, Sylvester Comprehensive Cancer Center, University of Miami, Miami, FL, United States; ^2^Hematology Research Center, Shiraz University of Medical Sciences, Shiraz, Fars, Iran

**Keywords:** cancer, outcome, survival, delay in diagnosis, disparities (health)

Delay in cancer diagnosis can result in a higher mortality rate and disease burden as well as a lower cure rate for cancer patients. Different factors can be related to the delay in cancer diagnosis including patient-related, healthcare system-related, and tumor-related factors. Our goal in this topic is to identify factors associated with the delay in diagnosis such as disparities and try to find applicable approaches to reduce them while investigating the factors affecting outcome. This can include diagnostic modalities, predictive models, and non-biologic factors affecting patients such as race, ethnicity, age, and patients' access to medical care and facilities. In the pandemic era, we faced extra new challenges which are also included in our discussion.

While diagnosis and management of cancer are time-sensitive, healthcare facilities were overwhelmed during the pandemic. The studies about the impact of the COVID-19 pandemic on cancer care can help the health systems and policymakers optimize the post-pandemic use of resources.

The impact of COVID-19 on outpatient visit volume in cancer patients was investigated by Frisardi et al. in a multicenter retrospective observational study. Although there was not any significant difference in the first pandemic wave, between COVID-free and COVID-mixed institutes and between the comprehensive cancer centers (CCCC) and community hospitals, later in 2021 the study showed a better outcome organizing COVID-mixed pathway in the CCCCs rather than to keep the Institutions COVID-free considering the patients' needs and convenience to cancer care.

Disparities can occur in different ethnic groups. According to Chen et al. American Indians have experienced an avoidance of medical care during the pandemic. The study identified more delays, cancelations, and medication inaccessibility to American Indians during the pandemic which could result in higher morbidity and mortality of cancer.

Besides the pandemic, there have been other factors affecting different patient groups for health care access. According to Dhir et al. in a study of adolescent and young adult (AYA) Lymphoma patients, young adults have the lowest rate of healthcare utilization. Some contributing factors include inadequate health coverage, lack of insurance, education, difficulties in transitioning from pediatric to adult care settings, and the lack of understanding in preventive health care measures. It ultimately can result in delays in diagnosis and advanced disease at presentation. Other factors affecting AYA group to seek lymphoma health care include the variability in therapeutic approaches based on age, location, and providers as well as access to therapeutic clinical trials which could overall have a negative impact on the outcomes of this patient population. Non-biological factors have been shown to impact the survival of cancer. Similar studies in older patients with lymphoma have indicated disparities in outcome due to factors such as insurance, income and socioeconomic status. The strong association of non-biological factors with survival sheds light on finding opportunities to improve the outcome and survival of AYA or any similarly vulnerable groups by improving their access to cancer care.

Finally, in this Research Topic, we included a systematic review and meta-analysis on cancer care pathway intervals and implications for survival in patients with oral cancer.

There have been mixed results in previous studies about patient outcomes in association with the intervals of the care pathway. The diagnostic and treatment intervals were studied by Fernandez-Martinez et al. to find the impact on tumor stage at diagnosis and survival. The three studied intervals include the following: “the patient (first symptom to first presentation to a healthcare professional), diagnostic (first presentation to diagnosis), or treatment (diagnosis to start of treatment) intervals in adult patients diagnosed with primary oral cancer.” This study revealed the influence of socioeconomic status as a key factor in interval duration and implications for patient outcomes. Countries with a lower income have been shown to have longer treatment and patient intervals which were related to a later stage at diagnosis. Further studies are needed for evaluation of survival.

Besides the great efforts in retrospective reviews of the disparities and factors affecting cancer outcomes, there is a need for further prospective cancer studies such as lymphoma epidemiology of outcomes cohort which can reduce the different potential biases and limitations of previous retrospective studies. Such studies can improve the pathway of understanding different epidemiologic implications on cancer patients with different ethnic, age, and socioeconomic groups. The patient's diagnosis and treatments, etc. in different regions and the outcomes of the patients can be studied on a large scale over time whether they went through the pandemic, dealt with post-pandemic effects, or at any other time. Meanwhile, the association of genetic and biological factors can be studied in these patients as well to clarify the role of non-biological factors that can be mediated and reduced in the setting of many different biological factors (1, 2).

We aim to continue monitoring the factors affecting cancer outcomes or delays in diagnosis while further studies are needed to clarify factors affecting outcomes and disparities in different parts of the world. To address these factors, it is important to promote cancer awareness and education, improve access and delivery of healthcare, and increase communication and collaboration within the healthcare system. Ensuring timely diagnosis and treatment can impact cancer outcomes and patient survival.

## Author contributions

SH: Conceptualization, Data curation, Investigation, Project administration, Supervision, Validation, Writing – original draft, Writing – review & editing. MD: Writing – original draft, Writing – review & editing. GD'A: Conceptualization, Supervision, Validation, Writing – original draft, Writing – review & editing.
